# Supplementary material for: Analysis of travel-time to HIV treatment in sub-Saharan Africa reveals inequities in access to antiretrovirals
Source: Commun Med (Lond). 2025 May 12;5:169. doi: 10.1038/s43856-025-00890-y (PMC12069690; doi:10.1038/s43856-025-00890-y)
Supplement: Supplementary file 1 — Supplementary Information [file 43856_2025_890_MOESM1_ESM.pdf]

Supplementary Information

Analysis of travel-time to HIV treatment in sub-Saharan Africa reveals inequities in access to antiretrovirals

Justin T. Okano, Andrea Low, Luckson Dullie, Wongani Mzumara, Harriet Nuwagaba-Biribonwoha, Sally Blower

Items included:
Supplementary Figure 1: Political maps of Eswatini, Malawi, and Zambia
Supplementary Figure 2: Comparison of sex-specific prevalence maps
Supplementary Table 1: Cross-validation metrics for the Empirical Bayesian Kriging (EBK) models

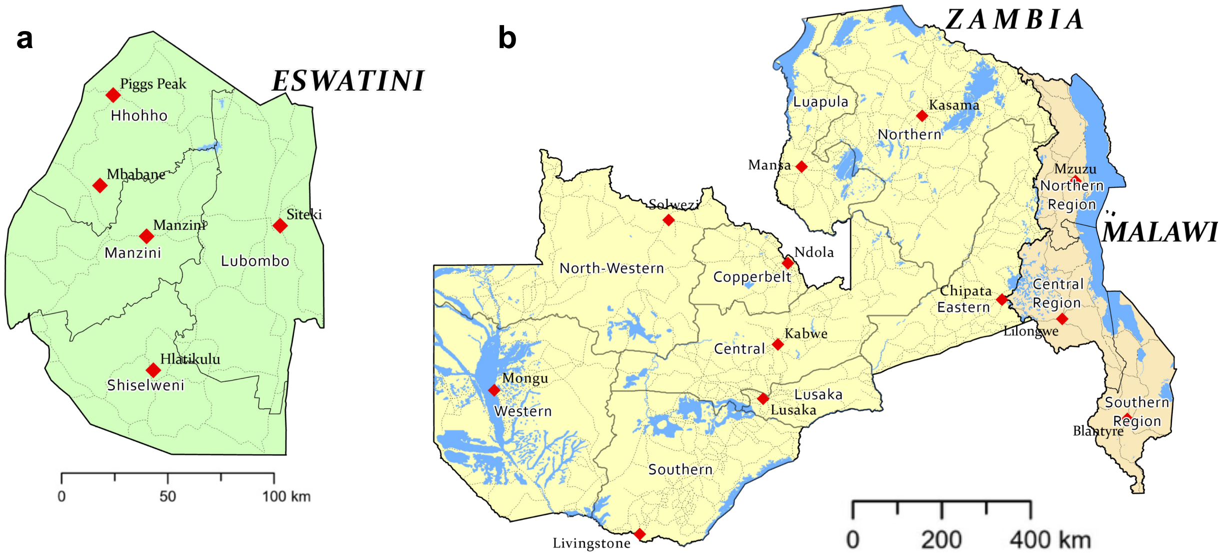

Supplementary Figure 1. Political maps of Eswatini, Malawi, and Zambia. Maps depict primary cities (red diamonds), level-1 administrative regions and boundaries (black lines), main roads (gray lines), and bodies of water (blue) for **a** Eswatini, and **b** Malawi and Zambia.

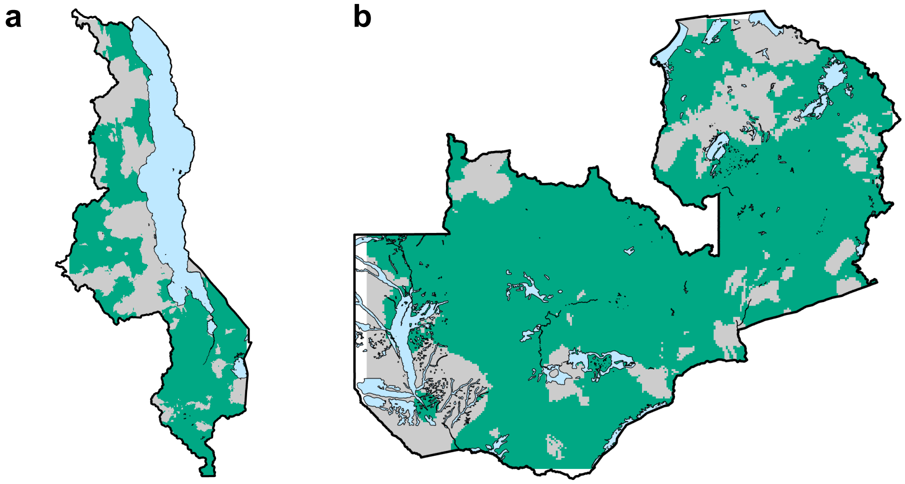

Supplementary Figure 2. Comparison of sex-specific prevalence maps. Maps comparing the ESP maps for women (Fig. 2b-c) with the ESP maps for men (Fig. 2e-f) for **a** Malawi, and **b** Zambia. Regions where predicted HIV prevalence in women is higher than predicted prevalence in men are shown in green; regions where predicted prevalence in men is higher than predicted prevalence in women are shown in grey. The predicted prevalence in women is higher than the predicted prevalence in men for all (100%) of Eswatini (not shown), 64% of Malawi, and 77% of Zambia.

	Eswatini			Malawi			Zambia		
	All	Female	Male	All	Female	Male	All	Female	Male
Mean Error	0.000	0.001	-0.001	0.001	0.001	0.001	0.000	0.001	0.001
RMS° Error	0.105	0.152	0.142	0.083	0.102	0.114	0.078	0.098	0.091
Mean Standardized	-0.003	0.011	-0.012	0.005	-0.006	0.008	-0.003	0.001	0.014
RMS° Standardized	0.961	0.979	0.966	0.977	0.995	1.023	1.005	1.008	1.004
Ave Standard Error	0.110	0.152	0.146	0.084	0.101	0.111	0.078	0.099	0.090
Average CRPS†	0.058	0.081	0.076	0.044	0.055	0.059	0.042	0.054	0.049
Percent in 90% PI‡	90.9%	89.2%	89.9%	90.0%	90.8%	92.2%	89.6%	91.6%	92.8%
Percent in 95% PI‡	95.8%	93.7%	95.1%	95.6%	95.4%	95.8%	95.7%	96.1%	94.9%

°Root Mean Square †Continuous Ranked Probability Score ‡Prediction Interval

Supplementary Table 1. Cross-validation metrics for the Empirical Bayesian Kriging (EBK) models. We used cross-validation to assess the quality of the EBK model predictions for Eswatini, Malawi, and Zambia. The resulting metrics show that the EBK models are properly configured and suitable for prediction. The 'Percent in 90% PI' and 'Percent in 95% PI' metrics indicate the percentage of measured values that are contained within 90% and 95% Prediction Intervals (these numbers should be close to 90% and 95%, respectively).
